# Analyses of transcriptomes and the first complete genome of *Leucocalocybe mongolica* provide new insights into phylogenetic relationships and conservation

**DOI:** 10.1038/s41598-021-81784-6

**Published:** 2021-02-03

**Authors:** Mingzheng Duan, Haiying Bao, Tolgor Bau

**Affiliations:** Key Laboratory of Edible Fungi Resources and Utilization (North), Ministry of Agriculture and Rural Affairs, Jilin Agricultural University, Changchun, 130118 Jilin China

**Keywords:** Fungal evolution, Fungal genomics

## Abstract

In this study, we report a de novo assembly of the first high-quality genome for a wild mushroom species *Leucocalocybe mongolica* (LM). We performed high-throughput transcriptome sequencing to analyze the genetic basis for the life history of LM. Our results show that the genome size of LM is 46.0 Mb, including 26 contigs with a contig N50 size of 3.6 Mb. In total, we predicted 11,599 protein-coding genes, of which 65.7% (7630) could be aligned with high confidence to annotated homologous genes in other species. We performed phylogenetic analyses using genes form 3269 single-copy gene families and showed support for distinguishing LM from the genus *Tricholoma* (L.) P.Kumm., in which it is sometimes circumscribed*.* We believe that one reason for limited wild occurrences of LM may be the loss of key metabolic genes, especially carbohydrate-active enzymes (CAZymes), based on comparisons with other closely related species. The results of our transcriptome analyses between vegetative (mycelia) and reproductive (fruiting bodies) organs indicated that changes in gene expression among some key CAZyme genes may help to determine the switch from asexual to sexual reproduction. Taken together, our genomic and transcriptome data for LM comprise a valuable resource for both understanding the evolutionary and life history of this species.

## Introduction

**Figure 1:**
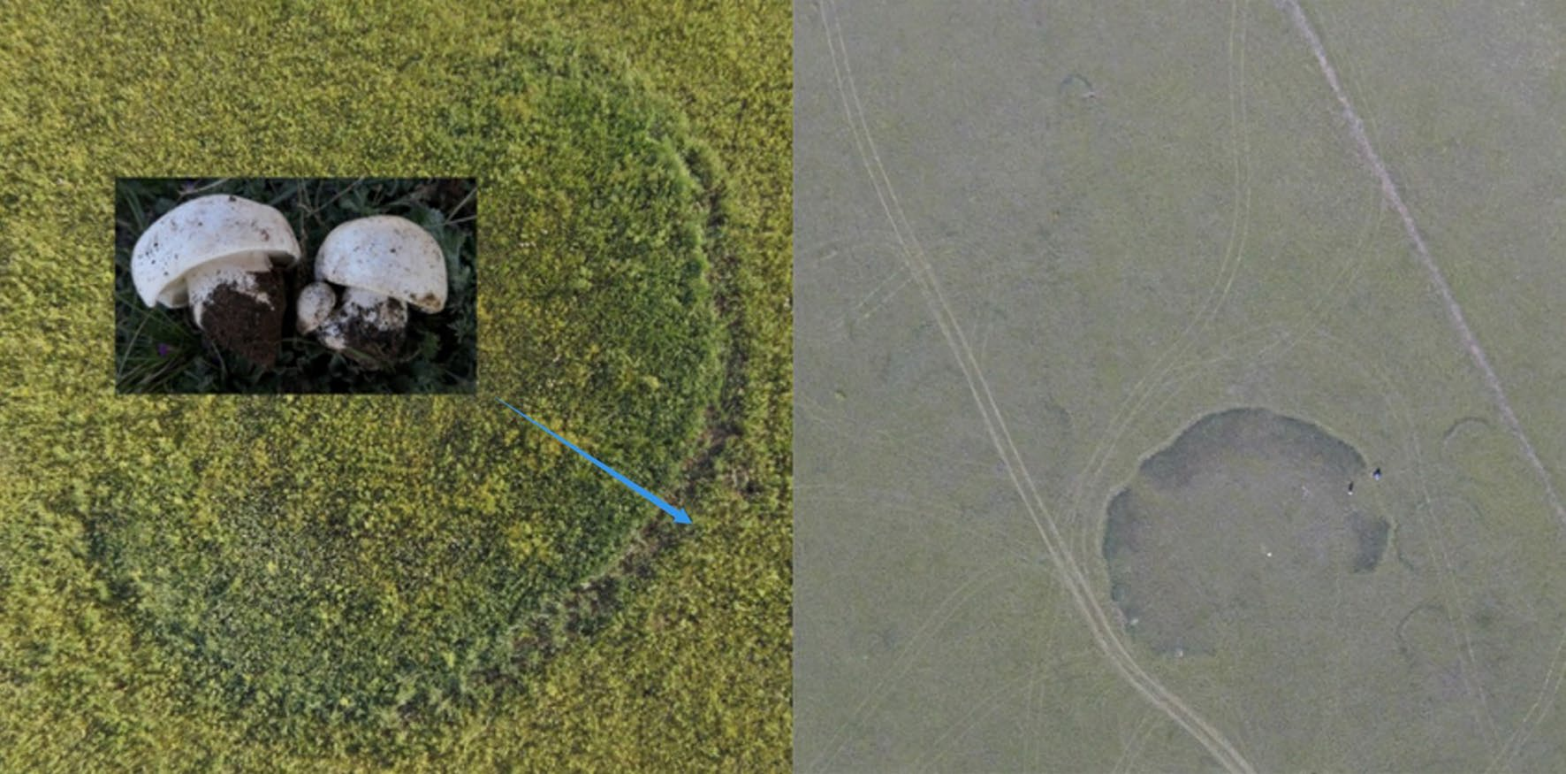
The fairy ring habit of LM in the Mongolian Plateau. The fruiting bodies grow on the outer edge of the ring.

*Leucocalocybe mongolica* (S. Imai) X.D. Yu & Y.J. Yao (LM) is a wild mushroom that is of food value throughout East Asia and for medicine on account of its natural pharmaceutical products, such as ergosterol, ergosterol peroxide, polysaccharide, and lectins^1–6^. Recently, Yu et al.^7^ excluded this species from genus *Tricholoma* (L.) P.Kumm. and assigned it to a new monotypic genus *Leucocalocybe* X.D. Yu & Y.J. Yao, based on morphological evidence and a phylogeny of the ribosomal large subunit rDNA (LSU). However, its treatment as a distinct genus has been controversial and some studies still used its old Latin Name^8–10^. LM is a species endemic in the Mongolian Plateau, where it forms fairy rings, a unique mycological growth pattern usually shown in grasslands^11^ (Fig. 1).

Presently, LM is threatened with extinction from the Redlist of China’s Biodiversity—Macrofungi (Reference number:000014672/2018–00,663) due to increasing drought in the Mongolian Plateau and over-collection for food and medicine. Since so-far limited success with LM cultivation, which could facilitate *ex-situ* conservation, we are not sure whether LM is a grass/wood-rot fungus or a mycorrhizal fungus. Prior conservation studies of LM have led to an improved understanding of its physiology, geographic distribution, and genetic diversity^12–14^.

Recently, with the rapid development of genome sequencing technology, high-resolution genomic sequences of many mushroom species have been reported^15–17^. The availability of these sequence data has facilitated comparative genomic analyses, especially among carbohydrate-active enzymes (CAZymes)^18–21^. Additionally, analyses of transcriptome data have led to the identification of important switches between vegetative and reproductive stages of species and of metabolic pathways that are active during each stage^22,23^. Consequently, genomic data have led to inferences of aspects critical to conservation, especially habitat preference, adaptability, and suitable cultivation conditions of mushroom species such as *Agrocybe aegerita* (V. Brig.) Vizzini^18^, *Sparassis crispa* (Wulfen) Fr.^19^, *Lentinula edodes* (Berk.) Pegler^24^, and *Hypsizygus marmoreus*.(Peck) H.E. Bigelow^25^, and within *Pleurotus* (Jacq. ex Fr.) P.Kumm^20,26,27^. In light of these prior studies, we believe that genomic resources can lead to breakthroughs in conservation efforts for LM, but genomic resources are currently largely unavailable for this species.

Therefore, in this work, we obtained a high-quality genome of LM using de novo assembly. We utilized the resulting dataset to reconstruct a phylogeny of LM and found that it is distinct from *Tricholoma*. Moreover, based on comparative genomic analyses, we detected deletion of several essential metabolic genes that may help to explain its rarity in the wild. Nevertheless, the composition of CAZymes in LM suggests potential domestications. Additionally, we observed that expression differences of oxidoreductase genes in LM appear to promote the transition from asexual to sexual reproduction. Taken together, these new genomic and transcriptomic resources of LM resulting from our study comprise a framework for future studies on taxonomy, genome function, and conservation of this species. Throughout, we follow the NCBI taxonomic database (REF) for taxonomic names except as noted.

## Results

### Genome sequence analysis

We sequenced genomic DNA from LM using the PacBio SMRT Sequel and Illumina platforms, and generated a total of ~ 228 × coverage (10.5 Gb, PacBio platform) and ~ 246 × coverage (11.35 Gb, Illumina platform) of high-quality data respectively (Table 1). The size of the assembled sequence of LM is 45.98 Mb, including 26 contigs with an N50 of 3.63 Mb and a GC content of 47.06% (Table 1). K-mer analysis based on Illumina reads indicated that the genome size was 47.69 Mb when K-mer was set at 21, while that was 44.67 Mb when K-mer was 35 (Supplementary Fig. S1), both of which were very close to the total length (45.98 Mb) of assembled genome sequence by using PacBio sequencing. In addition, the heterozygous ratio and the repetitive sequences of the LM genome were estimated to be 0.18% and 26.89%, respectively. We identified 95.2% (1271/1335) of well-known conserved fungal orthologs in this LM assembly using BUSCO^28^, suggesting a high-quality assembled mononuclear genome (Supplementary Table S1). Detailed genome statistics are shown in Supplementary Tables S1–S3 and Supplementary Figs. S2–S3. We performed genome annotation by de novo prediction and homology-based searches as well as a cDNA-based search using the transcriptome data generated in this study. In total, we predicted 11,599 protein-coding genes, which had a total length of 1835 bp and 6.3 exons on average (Table 2). Of these, we annotated 7630 (65.8%) genes (Supplementary Table S4) and functionally annotated 6855 (59.1%) genes according to the Gene Ontology (GO) database (Supplementary Fig. S4). Additionally, we performed annotations of repetitive sequences and non-coding RNAs (Table S5–S6), and found that 26.43% of the genome comprises repetitive sequences, while non-coding RNAs account for only 0.4% of the genome.

**Table 1:** General features of the LM genomic data.

Summary statistic	Value for LM genome
Genome size (bp)	45,983,313
Number of Contigs	26
Number of N50 Contigs	5
Contig N50 size (bp)	3,638,271
Number of N90 Contigs	12
Contig N90 size (bp)	866,782
GC content (%)	47.06
PacBio sequencing (depth)	10.5 Gb (228x)
Illumina sequencing (depth)	11.35 Gb (246x)

**Table 2:** Statistics of the genomes of LM and other Tricholomataceae.

Species	Gene number	Average gene length (bp)	Average CDS length (bp)	Average exons per gene	Average exon length (bp)	Average intron length (bp)
LM	11,599	1835.27	1427	6.3	226.72	77.89
LN	14,880	1700.07	1187	6.13	226.91	61.22
CG	19,049	1567.6	1078	6.04	204.97	66.21
LB	23,125	1549.22	1066	5.28	220.48	91.17
LA	17,553	1517.26	1092	5.12	241.96	68.87
TM	22,885	1205.98	821	4.03	234.9	86.4

### Comparative genomic and phylogenetic analyses

**Figure 2:**
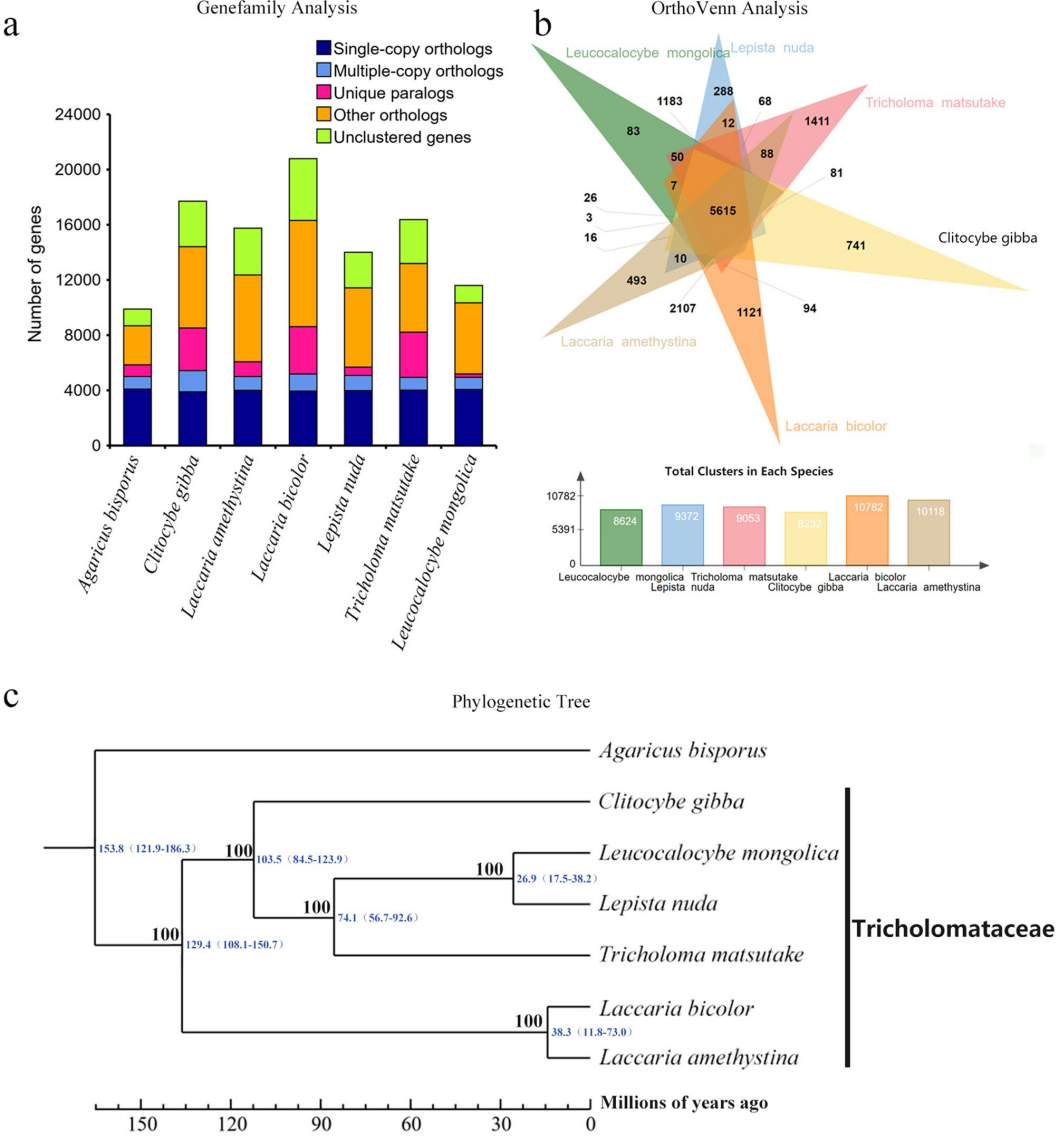
Phylogenetic and comparative genomic analyses of LM and five additional species of Tricholomataceae. (**a**) Distribution of gene clusters among species based on the GeneFamily approach. The horizontal axis represents species and the vertical axis is the number of genes. Single-copy orthologs refer genes present in single-copy in all species. Multiple-copy orthologs refer to genes present in multiple copies in all species. Unique paralogs refer to genes present in only one species. (**b**) Venn diagram showing the number of shared gene families among the six species of Tricholomataceae based on analysis using OrthoVenn. The lower figure shows the total number of clusters in each species. (**c**) Maximum likelihood (ML) phylogeny reconstructed from 3269 single-copy nuclear genes with dated nodes from TimeTree, the left side of nodes represent bootstrap index.

The dated phylogeny of Tricholomataceae (Fig. 2c) suggests that the common ancestor of the family diverged from a shared ancestor of *Agaricus bisporus* (J.E.Lange) Imbach (AB) ca. 153.8 million years ago (Mya), and *Tricholoma* began diversification about 103.5 Mya. *Leucocalocybe* and *Lepista* were resolved as sister species that diverged 26.9 Mya, about 74.1 million years later than their separation from *Tricholoma*, thus supporting the recognition of *Leucocalocybe* as a distinct genus rather than within *Tricholoma* (Fig. 2c).

We compared the genome of LM to five closely related species in Tricholomataceae: *Lepista nuda* (Bull.) Cooke (LN), *Tricholoma matsutake* (S. Ito & Imai) Singer (TM), *Clitocybe gibba* (Pers.) Harmaja (CG), *Laccaria bicolor* (Maire) P.D.Orton (LB), and *L. amethystina* Cooke (LA), and found that LM has the smallest number of genes among the sampled species (Table 2). Among all the six species including LM, we identified 15,817 gene families, of which 4389 occur in all the species and 3269 of them are single-copy gene families, which were used as gene markers to perform phylogenetic analyses in this study. However, LM exhibits a very low level of genetic differentiation with only 95 unique gene families compared to 221 in LN, which has the next lowest, and 989 in TM, which has the highest (Fig. 2a and Supplementary Table S7). This is particularly surprising given that TM is sister to a clade of LM and LN, thus highlighting the limited divergence in LM compared to the two most closely related species (Fig. 2c). Similarly, we identified 83 unique clusters of genes in LM, the smallest among all the species (Fig. 2b). The analysis by OrthoVenn also revealed that LM had the largest number of uniquely lost gene clusters (390) present in all the sampled species of Tricholomataceae except for LM (Supplementary Fig. S4). We annotated functions of the 390 gene families uniquely lost in LM according to the GO database. We found that most of the missing genes were mapped to biological processes, in which 93 genes matched biological process (GO:0008150) and metabolic process (GO:0008152), respectively, as shown in Supplementary Table S8. The analysis by OrthoVenn suggests that LM is experiencing gene loss at a higher rate than other species in the family. In fact, it is the only sampled member of the family that appears to be gaining genes at a slower rate than losing them (i.e., compare Fig. 2b and Supplementary Fig. S5). This unique evolutionary process in LM sets it apart from other Tricholomataceae and seems to offer additional support for its independence from *Tricholoma* as suggested by Yu et al.^7^*.*

### CAZymes of LM

**Figure 3:**
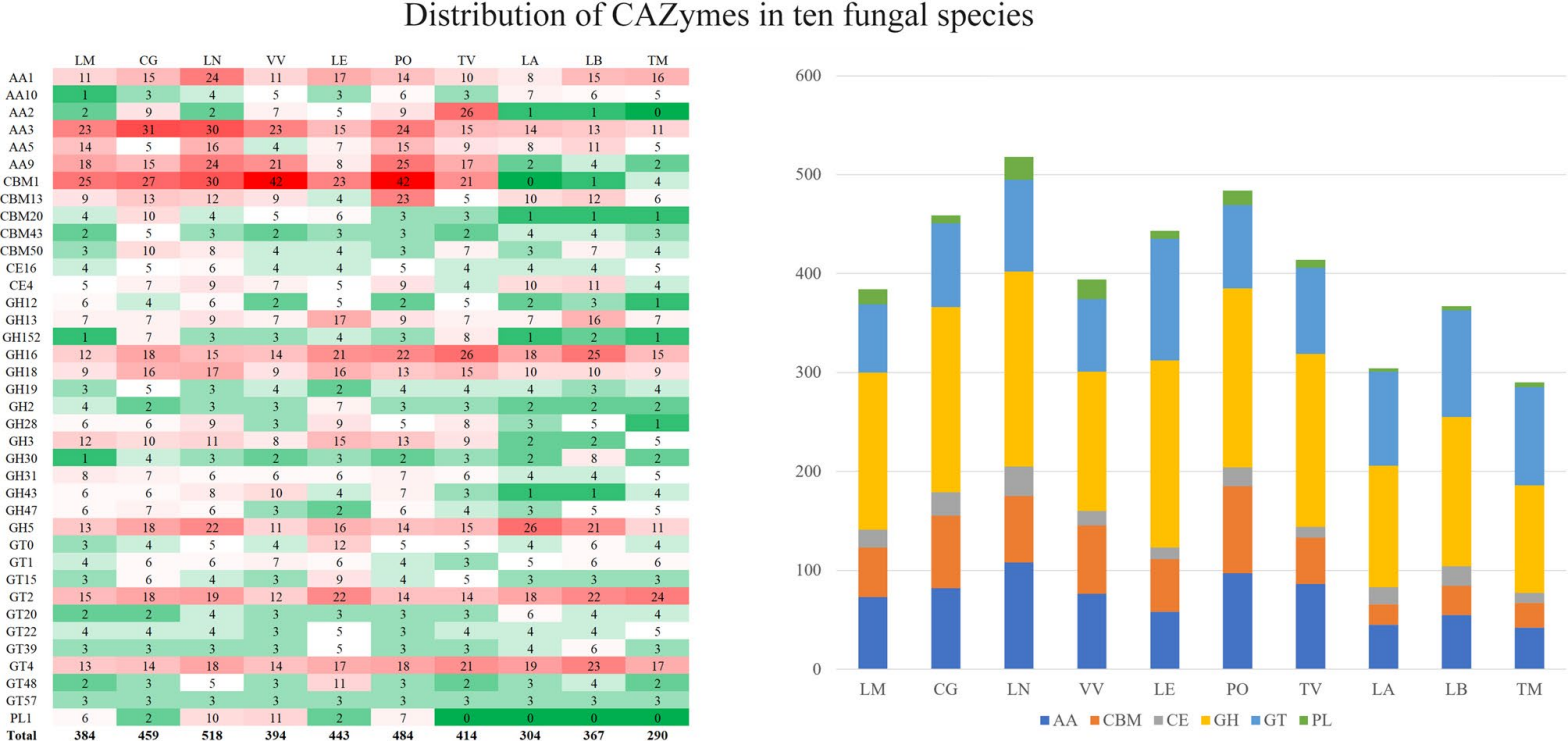
Statistics of CAZymes. (**a**) Heatmap showing the number of paralogs of CAZyme genes per species for families with an average of three or more paralogs among species. Warm colors represent higher numbers. Totals in the final row refer to the total numbers of genes per species. (**b**) Statistics for all CAZymes genes among the ten filamentous fungal species. The horizontal axis represents the species name and the vertical axis represents the number of CAZymes genes.

We sought to better understand the diversity of CAZymes in LM and thus the mechanisms of the species to metabolize carbon for nutrition. We annotated and compared all modules of gene families form CAZymes in LM with nine other fungal species including six grass- or wood-rot fungi. Of these, two species (CG and LN) were among those used in comparative genomic analyses; the other four species include *Volvariella volvacea* (Bull.) Singer (VV), *Lentinus edodes* (Berk.) Pegler (LE), *Pleurotus ostreatus* (Jacq. ex Fr.) P.Kumm. (PO), and *Trametes versicolor* (L.) Lloyd (TV). The remaining three species used for comparison are ectomycorrhizal fungi, which we also used in the comparative genomics analysis: TM, LB, and LA. In total, we annotated 384 CAZymes genes in LM, which is the smallest among the grass- and wood-rot fungi (Fig. 3 and Supplementary Table S9)*.* These genes were divided into six main modules corresponding to major CAZyme modules: 159 belonged to glycoside hydrolases (GHs, hydrolysis and/or rearrangement of glycosidic bonds), 71 were resolved to have auxiliary activities (AAs, redox enzymes that act in conjunction with CAZymes) and whose modules could code an important lignin degradation enzyme lytic polysaccharide monooxygenase (LPMO)^29^, 69 belonged to glycosyl transferases (GTs, formation of glycosidic bonds), 50 belonged to carbohydrate-binding modules (CBMs, adhesion to carbohydrates), 18 belonged to carbohydrate esterases (CEs, hydrolysis of carbohydrate esters), and 15 belonged to polysaccharide lyases (PLs, non-hydrolytic cleavage of glycosidic bonds) (Fig. 3). Notably, the number of CAZymes in LM detected in this study differed from our previous de novo transcriptome study^30^, in which we found 446 CAZyme genes in 6 modules. The difference may be attributed to unexpressed genes in the transcriptome data compared to our present analysis of the whole genome data. LM does not exhibit depletion or enrichment of any of the six CAZyme modules*.* However, in families of AA1, GH16, GH5, GT2, and GT4, LM has fewer genes than all the other grass- and wood-rot fungi.

### Transcriptome analysis of vegetative mycelia and fruiting bodies of LM

**Figure 4:**
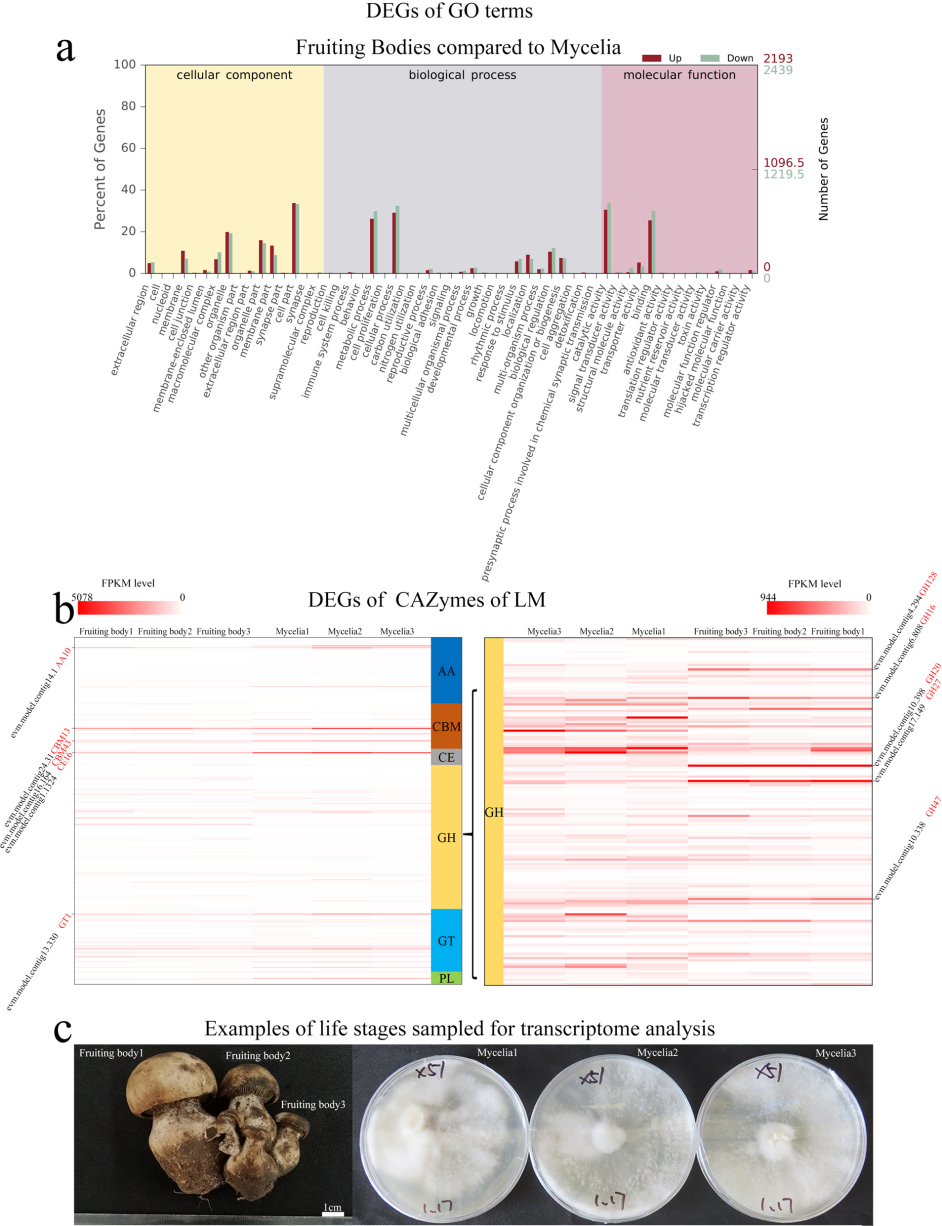
DEGs of GO terms and CAZymes. (**a**) GO term enrichment of the genes that are significantly up- or down-regulated in fruiting bodies. Gene name and its CAZyme family name. The right graph represents the genes within the GH module of CAZymes. Names to the right side indicate key gene names and their CAZyme family names. (**b**) Examples of fruiting bodies and vegetative (mycelia) organs of LM sampled for transcriptomic analysis.

In order to determine genes involved in fruiting body formation in LM (i.e., transition from vegetative growth), we performed high-throughput transcriptome sequencing of monocaryon mycelia (asexual stage) and fruiting bodies (sexual stage) with three biological replicates for each stage (Fig. 4c). In total, we obtained 449 million high-quality reads (average ~ 224 × depth per replicate per stage) and more than 80% of the sequencing data were properly aligned to the annotated exons (Supplementary Table S10 and Supplementary Fig. S6). Gene expression levels were estimated using FPKM (Fragments per Kilobase per Million Mapped Fragments) to compare between the two growth stages (Supplementary Table S11). Based on FPKM, the overall gene expression levels in fruiting bodies were higher than in mycelia (Supplementary Fig. S7a) in all replicate analyses (Supplementary Fig. S7c).

To determine differently expressed genes (DEGs) in LM, we compared gene expression among all samples and found that 2,192 genes in fruiting bodies were significantly up-regulated and 2,438 were significantly down-regulated. We used this result to build a volcano plot, which enabled us to visualize that the values of log_2_ fold change of most DEGs were within the range of ± 5 (Supplementary Fig. S7c. In addition, we found that the patterns of alternative splicing events between the two stages were slightly different (Supplementary Fig. S7d). Specifically, TTS (transcript start site) events occurred more frequently in mycelia compared with fruiting bodies, whereas IR (intron retention) events showed the opposite trend (Supplementary Fig. S7d).

To assess the enrichment of DEGs in fruiting bodies of LM compared with mycelia, we sought to detect enrichment for GO terms. (Fig. 4a) We found that DEGs in fruiting bodies were enriched in 16 terms representing biological processes (BP), 25 terms representing cellular components (CC), and 14 terms representing molecular functions (FC) (Fig. 4a). Among those, BP terms were primarily associated with metabolic processes, cellular processes, responses to stimuli, localizations and biological regulation; CC terms were mainly associated with the cell membrane, organelles and cell parts, and FC terms were related to catalytic activity and binding. We analyzed enrichment further according to expression levels and visualized the result using a Directed Acyclic Graph (DAG; Supplementary Fig. S8). Among CC terms, we found that genes annotated with the extracellular region (GO:0005576) and cytosolic part (GO:0044445) terms were enriched in the fruiting body stage. For BP, we found that carbohydrate metabolic process (GO:0005975) was greatly enriched in the fruiting body stage. For FC, iron ion binding (GO:0005506), cofactor binding (GO:0048037), catalytic activity (GO:0003824), and oxidoreductase activity (GO:0016491) were enriched in fruiting bodies.

We referred to genes related to fruiting body formation of *Lentinus edodes* and *Agrocybe aegerita*, but did not find similar expression in our transcriptome sequencing, which again proves that the mechanism of fruiting body formation of mushrooms is complex^22,31^. But notably, one BP term, carbohydrate metabolic process (GO:0005975), included most of the CAZymes, suggesting that some differentially expressed CAZyme may make significant contributions to the switch between vegetative and reproductive growth stages. All DEGs comprising CAZymes of LM are shown in Supplementary Tables S12–S13 and Fig. 4b. We identified 10 highly expressed CAZymes from the fruiting stage: five genes within the GH family, two in the CBM family, and three in the AA, CE, and GT families, respectively (Fig. 4b). Among these CAZymes there were (i) genes with expression levels that are equivalent in the two different stages (i.e., not DEGs), including AA1, CBM13, CBM43, CE16, and GT1, and (ii) genes that are up-regulated in fruiting bodies, including GH128, GH16, GH20, GH27, and GH47 (Fig. 4b). Based on their up-regulation in fruiting bodies, CAZymes of the GH family specifically may be involved in switching between reproductive stages in LM.

To verify the results from transcriptome data, we performed quantitative real-time PCR (qPCR) analysis on the 10 highly expressed CAZyme genes using the six materials shown in Fig. 4c. The results showed that the expression of all genes except for GT1 was consistent with that obtained from transcriptome analysis (Supplementary Fig. S9), indicating our transcriptome data are reliable.

## Discussion

### Taxonomy and phylogenetic relationships of LM

LM was firstly described by Imai Sanshi in 1917^32^ and included in the genus *Tricholoma*. More recently, Yu et al.^7^ separated LM from *Tricholoma* and proposed that the species is more closely related to *Lepista* based on morphology and a phylogeny of LSU DNA sequences. Yu et al. also argued that LM is morphologically distinct from *Lepista*, and according to our identification, the spore print of LM is white but that of genus *Lepista* should be more pink^33^. Based on these evidences, in combination with polyphyletic analysis, Yu et al. proposed a new genus, *Leucocalocybe*^7^. However, due to a single marker is used in Yu et al., the proposed taxonomic opinion remains controversial.

In our study, we used 3,269 single-copy genes to reconstruct a phylogenetic tree including six species of Tricholomataceae. The dated phylogeny presented here (Fig. 2c) reveals that LM is closer to *Lepista*, which is in agreement with the results of Yu et al. and our early study that LM diverged 25.5 Mya and *Tricholoma* diverged ca. 60 Mya. In addition, unique habitat (the fairy ring as shown in Supplementary Fig. 1), restricted distribution area (only grow in a small part of the Mongolian Plateau), and gene loss events also imply us that LM has a unique biological status. Collectively, these results support the conclusion of Yu et al. that LM belongs to the monotypic genus *Leucocalocybe*.

### Gene loss may be an intrinsic cause for rarity of LM in nature

In this study, we unexpectedly discovered several lines of evidence suggesting that LM has experienced extensive gene loss. Most notably, LM has the smallest number of genes among the six sampled species of Tricholomataceae (Table 2) as well as the smallest number of unique gene families (Fig. 2a), suggesting a low rate of differentiation and a high rate of gene loss. In addition, LM appears to have the highest number of uniquely lost gene families (i.e., absent in LM but none of the other five species) (Supplementary Fig. S5), supporting a high rate of gene loss instead of a slow rate of gene gain, though these are not mutually exclusive processes and both could work in this species. LM also has the smallest number of CAZymes among the six sampled saprophytic species (Fig. 3), suggesting that it has lost some key functions central to its carbon metabolism, thus, potentially affecting its ability to obtain nutrients. These intrinsic and evolutionary features of LM may be partially responsible for its rarity in nature.

### GO annotations and analyses of CAZymes represent genetic resources supporting cultivation of the species

In our study, we performed transcriptome sequencing on mononuclear mycelia and dikaryon fruiting bodies of LM, which represent the asexual and sexual stages of the species respectively. Our results for GO annotation of the two LM transcriptomes were consistent with prior studies on *Agrocybe aegerita*^34^, *Hypsizygus marmoreus*^25^, and *L. edodes*^24^, which indicate that GO terms related to the cell and cell membrane were the most common within the CC category, terms of catalytic activity and binding were the most common in the FC category, and terms of cellular processes and metabolic processes were the most common in the BP category (Fig. 4a). Additionally, we found the results of GO annotation in this study are very similar to our earlier de novo transcriptome study of LM^24^, which confirmed that we gain a reliable reference genome in this study.

Our analyses of GO terms between the two transcriptomes also revealed that carbohydrate metabolic processes (GO:0005975) genes are the most significantly enriched in fruiting bodies among DEGs (Supplementary Fig. S7). This seems to be in agreement with our findings based on CAZymes that acquisition and use of nutrients, particularly carbon sources, in LM may play a critical role in shaping the observed evolutionary and ecological patterns (fairy rings) and processes in the species.

Nutrient acquisition in LM is poorly understood. We compared CAZymes in grass- and wood-rot fungi (LM, LN, CG, VV, LE, PO, and TV) and symbiotic mycorrhizal fungi (TM, LA, and LB) and found that the symbiotic mycorrhizal fungi had fewer CAZymes, especially for the CBM1 family (Supplementary Table S9). n contrast to mycorrhizal fungi, the distribution of CAZymes in LM is more similar to grass- and wood-rot fungi, despite that LM has the smallest number of CAZymes among the grass- and wood-rot fungi. If the CAZymes in LM suggest similarity to grass- and wood-rot fungi in terms of nutrient acquisition modes, then LM may have high potential to be domesticated because many grass- and wood-rot fungi are relatively easy to domesticate because they do not require a living host.

We examined ten CAZyme genes in detail that were highly expressed within mycelia and fruiting bodies (Table 3). Among these genes, evm.model.contig14.1 (AA10), evm.model.contig24.31 (CBM13), evm.model.contig16.164 (CBM43), and evm.model.contig13.330 (GT1) were highly expressed in both organs, and they play important roles in the metabolism of carbon nutrients. Interestingly, three of these five genes belong to small-size gene families: evm.model.contig14.1 is the only member of the AA10 family in LM, evm.model.contig16.164 represents one of two total genes in the CBM43 family, and evm.model.contig13.330 is one of four total genes in the GT1 family. This indicates that the size of a CAZyme family is not predictive of its expression level. We also found that, of the ten highly-expressed CAZymes, five were related to metabolism of glucose, xylose, galactose, or mannose, which were previously deemed important for nutrition in LM^14^. They are evm.model.contig13.330 (GT1, glucose and xylose), evm.model.contig4.294 (GH128, glucose), evm.model.contig6.808 (GH16, xylose), evm.model.contig17.149 (GH27, galactose), and evm.model.contig10.338 (GH47, mannose). Therefore, these ten genes may play important roles in the nutrient metabolism of LM, and could thus affect fruiting body formation and have implications for domestication and breeding. Extraordinarily, we found the expression of CAZyme family GH128 (evm.model.contig4.294) was very high in the fruiting body group (Table 3), which was confirmed by the qPCR result (Supplementary Fig. S9). GO annotation (See the Supplementary Table S11) reveals that this gene (evm.model.contig4.294) participates in fungal-type cell wall polysaccharide metabolic process (GO:0071966). Moreover, by comparing against the NCBI Taxonomy database^35^, we found that this gene is widely distributed in many fungi, such as *Aspergillus nidulans* and *Neurospora crassa*^35^. Our study was the first to reveal and verify the high expression of the GH128 family gene in the fruiting bodies of LM, which promotes the research on the *ex-site* conservation, and also sheds light on the research of fruiting body formation of the other mushrooms.

**Table 3:** Candidate CAZymes, which show the greatest differential expression between fruiting and vegetative stages of LM, and their annotations.

Gene ID	CAZY family	Log2fold change	CAZy annotations
evm.model.contig14.1	AA10	− 0.44	AA10 (formerly CBM33) proteins are copper-dependent lytic polysaccharide monooxygenases (LPMOs); some proteins have been shown to act on chitin, others on cellulose
evm.model.contig24.31	CBM13	− 0.44	Modules of approx. 150 residues which always appear as a threefold internal repeat
evm.model.contig16.164	CBM43	− 0.98	Modules of approx. 90–100 residues found at the C-terminus of GH17 or GH72 enzymatic modules and also sometimes isolated
evm.model.contig1.1324	CE16	− 1.37	Acetylesterase (EC 3.1.1.6) active on various carbohydrate acetyl esters
evm.model.contig13.330	GT1	− 1.26	UDP-glucuronosyltransferase (EC 2.4.1.17); zeatin O-beta-xylosyltransferase (EC 2.4.2.40); 2-hydroxyacylsphingosine 1-beta-galactosyltransferase (EC 2.4.1.45)
evm.model.contig4.294	GH128	4.55	Beta-1,3-glucanase (EC 3.2.1.39)
evm.model.contig6.808	GH16	2	Xyloglucan:xyloglucosyltransferase (EC2.4.1.207); keratan-sulfate endo-1,4-beta-galactosidase (EC 3.2.1.103); endo-1,3-beta-glucanase (EC 3.2.1.39)
evm.model.contig10.398	GH20	4.12	Beta-hexosaminidase (EC 3.2.1.52); lacto-N-biosidase (EC 3.2.1.140); beta-1,6-N-acetylglucosaminidase (EC 3.2.1.-)
evm.model.contig17.149	GH27	2.95	Alpha-galactosidase (EC 3.2.1.22); alpha-N-acetylgalactosaminidase (EC 3.2.1.49); isomalto-dextranase (EC 3.2.1.94)
evm.model.contig10.338	GH47	2.75	Alpha-mannosidase (EC 3.2.1.113)

The gene IDs represent name of predicted protein-coding genes in LM.

## Conclusion

We sequenced, annotated, and studied the first whole genome of LM as well as transcriptomes representing its vegetative and fruiting organs. Our study may help to facilitate conservation of this rare species by suggesting a genomic basis for its rarity in the wild and by providing specific genomic resources for its domestication for food and medicine. In particular, we found that ten key CAZymes are associated with nutrient acquisition and sexual reproduction in LM and could be utilized for rapid advancement in its domestication, which represents an important method of ex situ conservation.

## Methods

### Materials

The s29 strain of LM was isolated from a spore print of a specimen acquired in the fall of 2018 in Chenbaerhu Banner of Hulunbeier City, Inner Mongolia Autonomous Region, China. The voucher specimen is deposited in the Herbarium of Mycology of Jilin Agricultural University (HMJAU), under NO. 55229. We verified separation of the monospore strain using an Axio Imager A2 fluorescence microscope (Zeiss). We cultured the monokaryotic mycelia in potato dextrose and carrot sucrose solid-state (potato 100 g, carrot 100 g, and sucrose 20 g per liter) culture in the dark at 23 °C for 20 d. The fruiting body for transcriptome sequencing was acquired in the fall of 2017 in Wubuer Baolige Sumu of Hulunbeier City, Inner Mongolia Autonomous Region, China. Then, we selected three samples from a tufted wild fruiting body (Fig. 4c) as biological duplications to guarantee the same growth conditions and stored in a − 80° freezer prior to use, and all sequencing strains and materials were stored in Engineering Research Center of Chinese Ministry of Education for Edible and Medicinal Fungi, Jilin Agricultural University. For collection ethics and protection of the species, we neither collect fruiting bodies of LM less than two in pre fairy ring, nor collect fruiting bodies that have not yet begun to eject spores. Finally, we leave at least one mature fruiting body in each fairy ring after collection.

### Isolation of RNA and construction of cDNA library

Total RNA was extracted from frozen mycelium and internal tissues of fruiting body by using the Transzol plant kit (TransGen Biotech, Inc.) following the manufacturer’s instructions. After extraction and purification, we checked the purity of RNA using a K5500 spectrophotometer (Kaiao, Beijing, China) and determined the integrity of RNA and its concentration with an RNA Nano 6000 Assay Kit of the Bioanalyzer 2100 system (Agilent Technologies, CA, USA). A total amount of 2 μg RNA per sample was used as input for the RNA sample preparations. We generated sequencing libraries with a NEB Next Ultra RNA Library Prep Kit for Illumina (#E7530L, NEB, USA) following recommendations of the manufacturer, and added index codes to attribute sequences to each sample. Briefly, we purified mRNA from total RNA using poly-T oligo-attached magnetic beads, and carried out fragmentation using divalent cations under elevated temperature in NEB Next First Strand Synthesis Reaction Buffer (5 ×). We synthesized the first strand of cDNA using a random hexamer primer and RNase H, and the second strand using buffer, dNTPs, DNA polymerase I, and RNase H. We purified the library fragments with QiaQuick PCR kits and performed elution with EB buffer.

### DNA extraction, library preparation, and LM genome sequencing and assembling

(a) Genomic DNA was extracted from strain s29 using DNeasy Plant Mini Kit (QIAGEN). Sequencing was carried out on the Pacific Bioscience Sequel platform and Illumina platform at Annoroad Gene Technology Company, China. In total, we generated 10.5 Gb of high-quality reads from the SMRT cells and 11.35 GB of high-quality paired-end reads from the Illumina platform. (b) Assembly: We assembled the PacBio reads using the Mecat pipeline^36^, and curated the assembled contigs using the Arrow algorithm^37^. We curated the data from Illumina platform using Pilon^38^. We used BUSCO^39^ with OrthoDB database^40^ to assess the integrity of the assembled genome sequence.

### Transcriptome analysis

We sequenced mRNA from monokaryotic mycelia and fruiting bodies, we performed 3 replicates for each group, on the Illumina Hiseq 2500 platform (Illumina; San Diego, CA, USA). We aligned the resulting high-quality pair-end reads to the assembled LM genome sequence using HISAT2 v2.1.0^41^ with default parameters, and applied StringTie v1.3.2d for transcriptome assembly with default parameters. We used HTSeq (0.6.0)^42^ to calculate expression with parameters “-i gene_id -f bam -s no -a 10 -q”, and identified DEGs with DEGseq v1.18.0. We estimated up/down-regulated genes at conditions of |log_2_ fold change|> 2, *p*-value = 0.05, and *q*-value = 0.05. We applied Asprofile v1.0.4 (http://ccb.jhu.edu/software/ASprofile/) to identify alternative splicing (AS) events.

### Annotation of the LM genome

(a) Gene prediction: We performed genome annotation by de novo prediction and homology-based searches as well as a cDNA-based search using the transcriptome data generated in this study. We used Augustus v3.3^43^, SNAP^44^, GeneMark v4.33^45^, and GlimmerHMM v3.04^46^ to predict gene sequences and calculate codon frequency and exon/intron distribution. Our homology-based searches comprised aligning predicted genes in LM to sequences in eggNOG, Pfam, and the NR and NT databases of the NCBI following methods in Yuan et al^21^. We used Blastp v2.2.28 (version used throughout unless otherwise noted)^47^ to compare the annotated protein-coding sequences in LN, TM, CG, LB, and LA to LM s29. For transcriptomes, we used Tophat v2.1.1^48^ to determine splice junctions and PASA v2.10^49^ to generate the annotations. Finally, we used EVidenceModeler^50^ to combine all the above annotation results into one non-redundant annotation list. (b) Gene functional annotation: We used Blastp to compare our sequencing data for LM with annotated, curated sequences in SwissProt^51^, the National Center for Biotechnology Information (NCBI) nr and nt databases, GO^52^, NCBI Clusters of Orthologous Groups of proteins (COG)^53^, KO (KEGG Kyoto Encyclopedia of Genes and Genomes)^54^, and Pfam^55^ databases. We extracted functional information from the results according to the types of data available in each database. (c) Non-coding RNA annotation: We used Blast to compare our sequencing data with the Rfam^56^ database to identify rRNAs, snRNAs, and miRNAs, and used tRNAscan-SE v2.0.2 to determine tRNAs in our dataset.

### Comparative genomics and phylogenetic analyses

(a) Gene family identification: A GeneFamily approach^57^ was conducted. Briefly, we first filtered the gene set of protein sequences of LM, AB, CG, LN, TM, LA, and LB based on filtration standard, that is, when a gene has more than one transcript, the longest transcript is taken, and the protein sequences with lengths greater than 50 amino acids (aa) were picked. Second, we used Blastp to format the filtered data with parameters "-p blastp -m 8 -e 1e-5 -a 10 -F F" and then OrthoMCL^58^ software with the parameter " -I 1.5" to statistical gene family data form formatted data. (b) Phylogenetic analyses: By using results from gene family identification, we found 3,269 single-copy gene families in LM, AB, CG, LN, TM, LA, and LB genome data. Then, we used MUSCLE^59^ to generate a super-alignment of 3,269 single-copy gene families and reconstructed a phylogenetic ML tree by PhyML v3.0^60^. The HKY85 model was used and the bootstrap values were calculated with 100 replicates. (c) Molecular clock analysis: We used the time correction points from TimeTree^61^ of Life to infer the divergence times of phylogenetic analysis, including *Laccaria bicolor* and *Agaricus bisporus* at 111–189 Mya, *Agaricus bisporus* and *Tricholoma matsutake* at 111–189 Mya, *Laccaria bicolor* and *Tricholoma matsutake* at 107.6–151.1 Mya (divergence times were obtained on 2019.4.9). The MCMCtree in PAML^62^ and the BRMC method^63^ were used to estimate the divergence time, with the time correction points obtained from TimeTree^61^. (d) OrthoVenn2^64^: We performed analyses in OrthoVenn2 with an e-value <  = 1e-15 and an inflation value = 1.0. (e) Reference genome sources: All reference genome sequences used in this study were retrieved from Joint Genome Institute (JGI; https://genome.jgi.doe.gov/), and are list in Supplementary Table S13.

### Identification of CAZymes

We first downloaded the CAZy enzyme database^65^ (http://bcb.unl.edu/dbCAN2/download/CAZyDB.07312019.fa as database, and http://bcb.unl.edu/dbCAN2/download/Databases/CAZyDB.07312019.fam-activities.txt as the annotation file). Then, we performed a Blastp search to align CAZymes of LM and other 9 species identified in this study to the CAZyme database; the top hits with e-value <  = 1e-17, minimum homology rate > 45%, and coverage > 45% were considered to be homologs.

### qPCR nalysis

qPCR was performed using the same DNA samples and primers described in Fig. 4c. The qPCR reaction conducted in a 15-μL volume containing 2 μL AceQ qPCR SYBR Green Master Mix (JZ121-02, Jizhenbio), 0.7 μL of each primer (10 μM), 100 ng (1 μL) of cDNA templates, and ddH_2_O to a final volume of 15 μL. The qPCR cycling parameters were: 95 °C for 5 min, 40 cycles of 95 °C for 10 s and 60 °C for 30 s. The *Actin* gene was used as the internal control and the relative expression level of each gene was calculated by the 2^-ΔΔCt^ method. Each qPCR reaction was performed in triplicate. All of the primer sequences used are shown in Table S14.

## Supplementary Information

Supplementary Information 1.

## Data Availability

The whole-genome sequencing data for *L. mongolica* have been deposited into the NCBI BioSample database under accession number JAAXNY000000000 and PRJNA623488.
